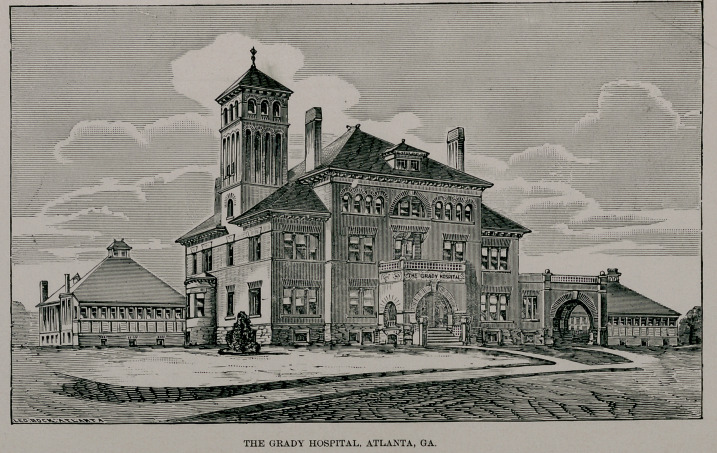# The Grady Memorial Hospital

**Published:** 1892-05

**Authors:** 


### *EDITORIAL.*

The office of THE JOURNAL is in rooms 44 and 45 Old Capitol Building.
Address communications relating to the Editorial Department to DR. L. B. GRANDY, Atlanta, Ga., Box 431.
Business communications should be addressed to DR. M. B. HUTCHINS, Atlanta, Ga.
All remittances should be made payable to "ATLANTA MEDICAL AND SURGICAL JOURNAL."
Articles for publication should reach this office not later than the 15th instant.
The editors are not responsible for views expressed by contributors.

### THE GRADY MEMORIAL HOSPITAL.

It is a little strange that the city of Atlanta, charitable as it is, should be only just now obtaining a city hospital for the treatment of the sick poor. For many years the desirability, or rather the necessity, of such an institution has been recognized, and a few years ago a movement was made in this direction. A meeting of citizens, which, it appears, has become historical in Atlanta annals, was held in the court-house, the usual speeches made, resolutions passed and committees appointed, etc. From the laudable desire to have the constitution of said committees non-partisan, impartial and representative, the necessary unities and harmonies were not attained, and the hospital movement "died à-bornin'."

About two years and a half ago a number of Atlanta's public spirited citizens renewed their efforts, determined to push the matter forward to a successful termination. A certain amount of work had been accomplished when the city sustained the loss of her favorite son, the late Henry W. Grady, Christmas, 1889. Mr. Grady had a warm place in the hearts of the people everywhere, and his fellow-townsmen, who knew him best and loved him most, determined to erect this monument to his memory. The suggestion to call it the "Grady Hospital" met with universal

THE GRADY HOSPITAL, ATLANTA, GA.

approval, and men and women, at home and abroad, gladly contributed their means and their energies to the good cause. From that time to the present the work has gone interruptedly, but surely, on, and at this writing there only remain a few strokes of the carpenter before the doors will be thrown kindly open to all who may need to enter.

The hospital has been built entirely by private subscription, principally by the friends and admirers of Mr. Grady in Atlanta and Georgia, and it is a fact worthy of note that every cent subscribed was collected. The largest contributor was the late Mr. W. A. Moore, who bequeathed to it seven thousand, five hundred dollars. The entire cost of all buildings and grounds will be about ninety thousand dollars.

Facing this editorial is an imperfect picture of the hospital as it stands completed. The hospital occupies a very pretty site of nearly four acres, and thus has ample room for subsequent additions, as these may become necessary. The main building is of Gothic structure, and in the rear are the pavilions and necessary accessory buildings. There are two wards for white patients and two for colored. No attempt will be made, as yet, to divide the wards into medical, surgical, gynecological, etc., but this will be done when the necessities of the institution require it. The hospital is essentially modern in all its appointments. The very latest and best systems of plumbing and ventilation have been employed regardless of expense, and it may be safely said that these are as near perfect as can be attained. The doors will be opened in May with facilities for the treatment of sixty ward patients. Arrangements are also provided for pay-patients, in private rooms, and as many as fifteen or twenty of these can be accommodated. The medical colleges of the city will derive great benefit from the clinical facilities afforded by the hospital. Students of the colleges, upon the payment of a small fee, will be entitled to attend all the clinics in the hospital during the collegiate year.

The management of the hospital is vested in a Board of Trustees, composed at present of Mr. Joseph Hirsch, Chairman, Mayor W. A. Hemphill, *ex officio*, Mr. S. M. Inman, Mr. T. B. Neal, Capt. R. J. Lowry, Mr. J. W. English, Dr. R. D. Spalding, Col. John T. Glenn, Mr. Jacob Elsas and Mr. W. L. Moore. These gentlemen have freely given their time and their money to this great charity, and the result of their faithful and untiring energies stands to-day as a monument to them also, as well as to him whose name it bears. Without making invidious comparisons, it may be said that to Mr. Joseph Hirsch belong especial honor and praise for the activity and fidelity which he has displayed in the good work.

The attending staff of physicians will be elected annually. The present staff is thus composed:

Physicians—Doctors J. F. Alexander, J. S. Todd, Henry Bak, C. G. Giddings, R. B. Ridley, W. S. Kendrick, J. G. Earnest.

Surgeons—Doctors Hunter P. Cooper, W. S. Elkin, W. P. Nicolson, W. S. Armstrong.

Oculists—Doctors A. W. Calhoun, A. G. Hobbs.

Gynecologists—Doctors Virgil O. Hardon, G. H. Noble.

The resident staff will be composed of the house physician and his senior and junior assistants.

The above represents about all that we started out to say with reference to the Grady hospital.

We have written hastily and in spite of frequent interruptions. The hospital, as it stands, is an ornament to the city, and a monument to one whose name Atlantians now delight to honor and perpetuate.

## Figures and Tables

**Figure f1:**